# Impaired Autophagy of GABAergic Interneurons in Neuropathic Pain

**DOI:** 10.1155/2018/9185368

**Published:** 2018-09-25

**Authors:** Yuhua Yin, Min-Hee Yi, Dong Woon Kim

**Affiliations:** ^1^Department of Medical Science, Chungnam National University School of Medicine, Daejeon 35015, Republic of Korea; ^2^Department of Anatomy and Cell Biology, Brain Research Institute, Chungnam National University School of Medicine, Daejeon 35015, Republic of Korea; ^3^Department of Neurology, Mayo Clinic, Rochester, MN 55905, USA

## Abstract

Neuropathic pain (NP) is caused by lesions of the peripheral fibers and central neurons in the somatosensory nervous system and affects 7–10% of the general population. Although the distinct cause of neuropathic pain has been investigated in primary afferent neurons over the years, pain modulation by central sensitization remains controversial. NP is believed to be driven by cell type-specific spinal synaptic plasticity in the dorsal horn. Upon intense afferent stimulation, spinothalamic tract neurons are potentiated, whereas GABAergic interneurons are inhibited leading to long-term depression. Growing evidences suggest that the inhibition of GABAergic neurons plays pivotal roles in the manifestation of neuropathic and inflammatory pain states. Downregulation of GABA transmission and impairment of GABAergic interneurons in the dorsal horn are critical consequences after spinal cord and peripheral nerve injuries. These impairments in GABAergic interneurons may be associated with dysfunctional autophagy, resulting in neuropathic pain. Here, we review an emerging number of investigations that suggest a pivotal role of impaired autophagy of GABAergic interneurons in NP. We discuss relevant research spurring the development of new targets and therapeutic agents of NP and emphasize the need for a multidisciplinary approach to manage NP in the future.

## 1. Introduction

More than 20 million individuals have neuropathic pain (NP) worldwide and suffer from highly acute diseases, greater healthcare costs, and lower quality of life [1–3]. In some cases, the most severe NP leads to increased episodes of depression and suicide [4–6]. The etiology of NP is broad and classified into central nervous system (CNS), peripheral nervous system, and complex neuropathic disorders, including complex regional pain syndrome types I (reflex sympathetic dystrophy) and II. Complex regional pain syndrome refers to a chronic pain condition characterized by progressively worsening spontaneous regional pain in the absence or presence of peripheral nerve damage. The traditional treatments for chronic pain, such as medications, massage therapy, acupuncture, electrical stimulation, nerve blocks, and surgery, cause severe side effects and fail to reduce or eliminate NP [7]. Further research is needed to understand the mechanisms underlying NP to allow for the design of individual and rational treatment strategies but also for the development of optimal therapeutic drugs.

Changes in the properties of neurons in the CNS can result in central sensitization. Even normal inputs that usually evoke innocuous sensations can lead to pain hypersensitivity due to the changes in the sensory response [8]. Moreover, there is evidence that central sensitization refers to subsequent peripheral nerve injury (PNI) or neuropathy [9]. In the past, many researchers believed that NP following PNI was due to the changes in the CNS and peripheral nervous system (PNS) neurons and their functions. Accordingly, many studies have focused on spinal dorsal neurons to elucidate the mechanism of NP. These studies have suggested that NP is a consequence of the aberrant response of the sensory neurons in the dorsal horn to the PNS inputs, anatomical reorganization of the pain pathway, as well as attenuated microglial activation and inhibitory processes [10]. A number of mechanisms have been suggested for central sensitization, among which there is strongest evidence for the dysfunction of GABAergic interneurons and their role in modifying synaptic transmission of pain signaling pathways in response to PNI [11–13].

Under normal circumstances, inhibitory interneurons continuously release GABA to decrease the excitability of lamina I output neurons and modulate pain transmission. However, this inhibition can be lost after an injury, resulting in hyperalgesia [10, 14]. This may occur as a result of changes in loss of GABAergic function or apoptosis. Additionally, disinhibition may enable nonnociceptive myelinated A*β* primary afferents to engage the pain transmission circuitry, leading to a perception of pain in patients in response to normally innocuous stimuli. It remains highly controversial to link the changes of cell types and cell death in the spinal dorsal horn with NP. It was reported that partial PNI leads to neuronal cell death in the dorsal horn [15]. Spinal cord injury-induced GABAergic interneuron cell death and their decreased number in the dorsal horn diminishes GABAergic tone and NP [14]. However, it has also been reported that the number of GABAergic interneurons and the synaptic boutons at GABA_A_ synapses, as well as the expression levels of intracellular GAD65, GAD67, GABA, GABA transporters 1 and 3, and GABA_A_ and GABA_B_ receptors can all remain unaltered [16–20].

The differences in these reports may be reconciled when considering time-dependent changes due to autophagy. Autophagy, a cellular self-digestion pathway involved in protein and organelle degradation, is known to be highly involved in various human diseases and physiology. Autophagy regulates cell homeostasis with a dual role in cell protection and cell death. However, some roles of autophagy are related to NP. We will discuss the importance of GABAergic neurons in NP and their regulation by autophagy in this review.

### 1.1. GABAergic Neuron Dysfunction in NP

Lamina II of the dorsal horn (substantia gelatinosa) is a major target of nociceptive primary afferents. Although excitatory and inhibitory interneurons show considerable heterogeneity, a morphological study reported that the islet cells are inhibitory, the radial and large vertical cells are excitatory, and some neurons with a morphological appearance of vertical cells are GABAergic [21]. It is estimated that approximately 30% of lamina II neurons are GABAergic, especially with enriched glycine. The rest appear to be glutamatergic and have high expression of vesicular glutamate transporter 2 [22–24].

GABAergic inhibition has been suggested to play a pivotal role in the manifestation of NP and inflammatory pain states [15], and GABAergic interneurons are densely innervated in lamina I giant project neurons [25]. As a result, NP occurs during pharmacological inhibition of spinal GABA_A_ and GABA_B_ receptor-mediated inhibitory transmission [26–28]. Furthermore, GABA_A_ receptor-mediated postsynaptic currents were observed to be inhibited after substantially reducing the primary afferents in the substantia gelatinosa of the spinal dorsal horn [15]. Moreover, GABA_B_ receptor-mediated inhibition was lost after spinal nerve injury, particularly in the central terminals of primary afferents, leading to allodynia or spontaneous pain behavior [18, 29].

Endoplasmic reticulum (ER) stress was shown to be related to cellular reactive oxygen species (ROS) production, followed by an impaired autophagic flux. This cascade of events contributes to central sensitization of GABAergic interneuron dysfunction [30–33]. In particular, ROS are involved in long-term depression of GABAergic interneurons in mice [11]. Scavenging ROS enhance GABA current, and applying hydrogen peroxide inhibits GABA current, indicating that GABA release is redox sensitive [12]. L4 and L5 bilateral dorsal rhizotomy increases extracellular concentrations of excitatory amino acids in the dorsal horn and reduces the sensory glutamate-induced decreases in the expression of GABA-synthesizing enzymes and presynaptic inhibition [34, 35]. Taken together, both autophagic impairment and ER stress contribute to dysfunction of dorsal horn GABAergic neurons in patients with NP. Therefore, regulating GABAergic neurons and inhibiting their function in the superficial dorsal horn of the spinal cord [15] are attractive therapeutic strategies developed to treat NP.

### 1.2. Autophagic Impairment in NP

There are several mechanisms of programmed cell death, including apoptosis and autophagy. Apoptosis involves a cooperation among caspases, whereas autophagic cell death involves (but not necessarily by) autophagic vacuolization [36]. Most previous studies focused only on apoptosis, but the study by Berliocchi et al. introduced the role of disrupted autophagy in an NP model using spinal nerve ligation (SNL) [37]. LC3II and Beclin 1 protein levels were found to be elevated after SNL. We also first reported that LC3 and Beclin 1 levels were significantly increased in the L4-5 spinal cord segment ipsilateral to the injured side in rats after SNL compared with the sham group [20]. In addition, the levels of LC3 and Beclin 1 were also significantly increased in GABAergic interneurons of the spinal dorsal horn following PNI [20]. Taken together, these studies suggest that autophagy is involved in the induction and maintenance of NP. Furthermore, a disturbance in autophagy renders GABAergic interneurons vulnerable to PNI or NP stimuli.

### 1.3. Inhibition of Autophagic Induction by 3-Methyladenine (3-MA) Reduces NP Behavior

Autophagy is an evolutionarily conserved catabolic process that involves degradation of cytoplasmic components. Autophagy involves multiple proteins and signaling pathways that can be divided into four distinct steps: (1) induction, (2) assembly and formation of autophagosomes, (3) docking and fusion with lysosomal membranes, and (4) degradation [38]. Autophagic flux is defined as a measure of autophagic degradation activity [39]. Therapeutic strategies that inhibit autophagy-induced cell death have the potential to identify novel and effective targeted treatments for NP.

**Figure 1:**
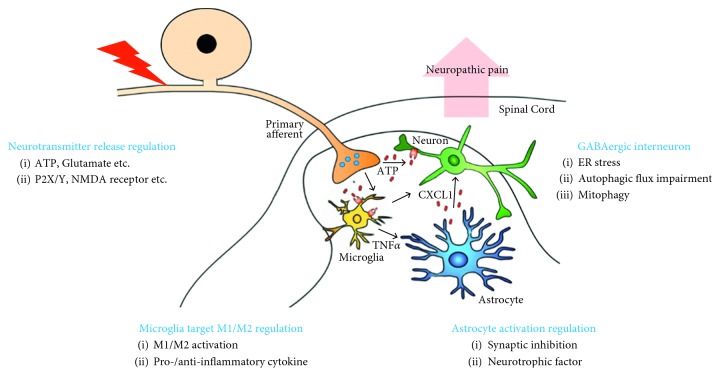
Therapeutic strategy to regulate GABAergic interneurons in spinal central sensitization. There is growing evidence that impaired autophagy and ER stress contribute to dysfunction of dorsal horn GABAergic neurons. Autophagosome accumulation and impairment of autophagic flux in GABAergic neurons make them susceptible to peripheral nerve injury or other pain stimuli. Therefore, there are several ways to control GABA neurons to control the spinal central sensitization that contributes to neuropathic pain. (1) Functional recovery of GABAergic interneurons, (2) neurotransmitter release regulation, (3) microglia targeted M1/2 polarization regulation, and (4) astrocyte targeted regulation.

3-methyladenine (3-MA) inhibits the induction of autophagy by blocking the formation of autophagosomes and inhibiting class III phosphatidylinositol 3-kinases [20, 40]. We injected 3-MA intrathecally to investigate whether inhibition of autophagic dysfunction would result in effective relief of NP. 3-MA treatment was found to significantly reduce mechanical allodynia in a time-dependent manner during postoperative observations before decreasing to baseline on day 14 (Figure 1) [20]. These results suggest that autophagy is a central regulator in the pathogenesis of NP.

### 1.4. Inhibition of Autophagic Flux by Chloroquine Reduces NP Behavior

Berliocchi et al. suggested that autophagy is modulated differently depending on the experimental pain model by demonstrating LC3I, Beclin 1, and p62 levels modulated differently for chronic constriction injury (CCI) versus spared nerve injury [41]. As described above, p62/SQSTM1 is an autophagic substrate and a key LC3-binding protein, which serves as a link between LC3 and ubiquitinated proteins [42]. p62 and p62-bound polyubiquitinated proteins become incorporated into the completed autophagosome and are degraded in autolysosomes. Because of the correlation between modulation of autophagy and p62 levels, this substrate is considered a useful proxy for autophagic degradation [42–44], and p62 levels are often increased when autophagy is impaired [43]. We previously demonstrated the increased accumulation of p62 on the ipsilateral side of CCI-induced mice compared with their contralateral side, suggesting that CCI leads to a blockage of the final degradative steps of autophagy [45].

We also reported that a high tissue content of omega-3 polyunsaturated fatty acids (PUFAs) attenuates formalin-induced pain sensitivity, microglial activation, inducible nitric oxide synthase expression, and phosphorylation of NR2B [46]. In addition, we also showed higher levels of LC3II and Beclin 1 but lower levels of p62 in fat-1 mice, suggesting that polyunsaturated fatty acid enrichment enhances the induction and flux of autophagy. In that experiment, we demonstrated that brain-derived neurotrophic factor (BDNF) is expressed at higher levels in fat-1 mice, suggesting that BDNF and its associated proteins Akt and CREB, may be therapeutic targets for NP, because BDNF might be a neuroprotective factor by potentially upregulating autophagic flux activity.

Finally, we investigated blocking autophagy using chloroquine. Chloroquine reverses autophagy by accumulating in the lysosomes and disturbing vacuolar H^+^ ATPase. This leads to a lysosomal acidification and results in autophagy inhibition [47]. Intrathecal injection of chloroquine into wild-type mice induces a significant reduction in the threshold of mechanical sensitivity [45]. Intrathecal chloroquine injection into naïve mice induces spinal accumulation of LC3 and p62, in parallel with significant mechanical hypersensitivity, thus confirming inhibition of autophagosome clearance and suggesting the participation of autophagy in spinal mechanisms of pain processing. Taken together, the analysis of LC3, Beclin 1, and p62 indicates that impaired autophagy in CCI-induced NP may result from blocking the late phase, rather than the induction phase of autophagic flux.

## 2. Mitophagy Impairment in NP

ROS are a major source of mitochondrial dysfunction and can induce mutations in mitochondrial DNA that result in protein deficiencies, restricted self-repair ability, and increase vulnerability of cells to ROS attack [48, 49]. Indeed, ROS induces oxidative stress, which damages mitochondrial proteins and lipids [50, 51]. In particular, mitochondrial oxidative damage is usually caused by aging and is the most important risk factor in most cases of mitochondrial dysfunction [52]. As a result, mitophagy counteracts against excessive intracellular ROS and blocks ROS sources, which are important in the therapeutic management of NP [53].

Mitophagy is the selective autophagic degradation of mitochondria that occurs when mitochondria become defective following a damage or stress [54, 55], such as hypoxia, nutrient starvation, energy depletion, and other pharmacological or viral stimuli [56]. Increased generation of mitochondrial ROS detected in L5 dorsal horn neurons of SNL-induced neuropathic rats in dorsal horn neurons [57]. PINK1 is a neuroprotective protein involved in the activation of mitophagy by selectively accumulating in depolarized mitochondria and promoting PARK2/Parkin translocation [58]. We examined the expression of PINK1 in the spinal cord and found the number of immunoreactive cells and PINK1 protein expression were both increased significantly on the ipsilateral and contralateral sides of the spinal dorsal horn in wild-type mice. However, no such significant differences were observed in toll-like receptor 4 (TLR4) knockout mice [45]. PINK1 is expressed in neurons in the spinal dorsal horn, but not in astrocytes or microglia [45]. Thus, we demonstrated that mitophagy may play a role in NP-related processes, but further studies are needed to clarify the function of mitophagy in NP, especially in GABAergic interneurons.

## 3. Impaired ER Stress in NP

ER stress is caused by disruption of the structure and function of the ER [59]. The unfolded protein response (UPR) can be induced by a variety of cellular stresses, such as glucose deprivation, depletion of ER Ca^2+^ stores, exposure to free radicals, and accumulation of unfolded or misfolded proteins [60, 61]. UPR is mediated by three ER stress receptors: PKR-like ER kinase, inositol-requiring enzyme 1, and activating transcription factor-6. Both ER stress and the resulting UPR are part of the cellular homeostasis program that balance the protein turnover and synthesis [62, 63]. However, if this equilibrium is disrupted, ER stress can activate programmed cell death pathways. Accordingly, activated ER stress pathways have been observed in various disease pathogenesis, such as those of diabetes, cancers [64, 65], Alzheimer's disease, Parkinson's disease, amyotrophic lateral sclerosis, and prion diseases [60, 66]. We also reported that the causes of ER stress in NP are related to autophagy and that autophagy was activated in an SNL-induced NP model [33].

As a common cytoprotective mechanism, autophagy controls homeostasis in various ways, such as protein and organelle turnover [67, 68]. Moreover, autophagy is associated with the ER at various molecular levels, and ER stress induces autophagy in mammalian cells via several canonical UPR pathways [69, 70]. We previously found that impaired ER stress in an SNL model and after ATF6 siRNA treatment significantly reduced NP-associated behaviors [33], indicating that ER stress is involved in the induction and maintenance of NP. Furthermore, we suggested that a disruption of the UPR pathway may render the neurons to become sensitive to PNI or NP stimuli. Further elucidation of the mechanisms of ER stress on GABAergic interneurons in the spinal cord during NP will be critical to guide the future development of novel pharmacological NP treatments.

## 4. Nonneuronal Cell Regulation of NP

In parallel with the progress achieved regarding these neuronal mechanisms, there has been an increased appreciation of the importance of nonneuronal cells, particularly glial cells such as microglia and astrocytes, to also play an important role in chronic pain. Over the past decade, there has been a dramatic increase in the number of pain research studies investigating the role of glia, which we briefly summarize in the following.

### 4.1. Activation of Spinal Microglia and NP

Microglia are resident macrophages of the spinal cord and brain. Although microglia comprise 5–12% of the cells in the CNS [71], abundant evidence supports their role in pathological pain [72–74]. Microglia are activated in the pathophysiological condition that occurs after PNI. [75] Once activated, microglia show morphological changes [76], increased expression of microglial markers, e.g., IBA1, MHC II, and CD 11b [76–78], increased numbers (proliferation) [79–81], and increased phagocytosis [82]. TLR4 was previously reported to be primarily expressed in microglia; it was found that neuronal TLR4 regulates the proliferation of neural precursor cells during axonal growth, adult neurogenesis, and neuronal plasticity [83]. We reported previously that neuronal cells, and not astrocytes or microglia, of the CCI-induced NP spinal dorsal horn had impaired autophagy [45]. Our data showed that chloroquine treatment in TLR4 knockout mice attenuated the pain threshold in CCI-induced mechanical allodynia compared with TLR4 wild-type mice. Therefore, it is reasonable to conclude that the proinflammatory cytokines release in microglia, and the impaired autophagy in neurons synergistically contribute to pain sensory hypersensitivity via TLR4, suggesting TLR4-mediated microglial activation may be indirectly coupled to autophagy.

### 4.2. Activation of Spinal Astrocytes

Increasing number of studies has shown that astrocytes are important in the development of chronic pain by regulating the extracellular concentrations of GABA released from neurons and glia by controlling its uptake [84–86]. Ischemia or hypoxia and gangliosides activate the autophagic/lysosomal pathway in astrocytes under oxidative stress [87]. Those authors reported that inhibiting this key system is protective. However, there have been no reports of activated autophagy in astrocytes of the spinal dorsal horn following NP or of autophagic impairment as an effective alternative treatment for NP. A tonic form of synaptic inhibition occurs in discrete regions of the CNS and has an important role in controlling neuronal excitability [88]. GABA in astrocytes is the major source of tonic inhibition in the cerebellum [89]. Although it is known that PNI alters astrocyte activity [90], it is unknown how astrocytes function as GABAergic and GABA-ceptive cells following PNI. Taken together, astrocytic GABA may actively participate in disinhibition of the neuronal networks in the spinal dorsal horn to regulate pain.

## 5. Conclusions

An accumulating body of literature has provided compelling evidence for the importance of neurons and glial cells in NP. The mechanisms regarding GABAergic interneuron dysfunction remain to be further elucidated. In this review, autophagic impairment of GABAergic interneurons in the spinal dorsal horn was identified as a new regulator of spinal neuronal circuits (Figure 1). We propose that impaired autophagy plays a critical role in spinal hypersensitivity, to which reduced GABAergic interneuronal activity is a key contributor in NP. This concept provides insight into the pathogenesis of NP and suggests potential strategies for developing new treatments for NP.
